# Supplementary figures and images for: The braingraph.org database with more than 1000 robust human connectomes in five resolutions
Source: Cogn Neurodyn. 2021 Mar 12;15(5):915–9. doi: 10.1007/s11571-021-09670-5 (PMC8448809; doi:10.1007/s11571-021-09670-5)

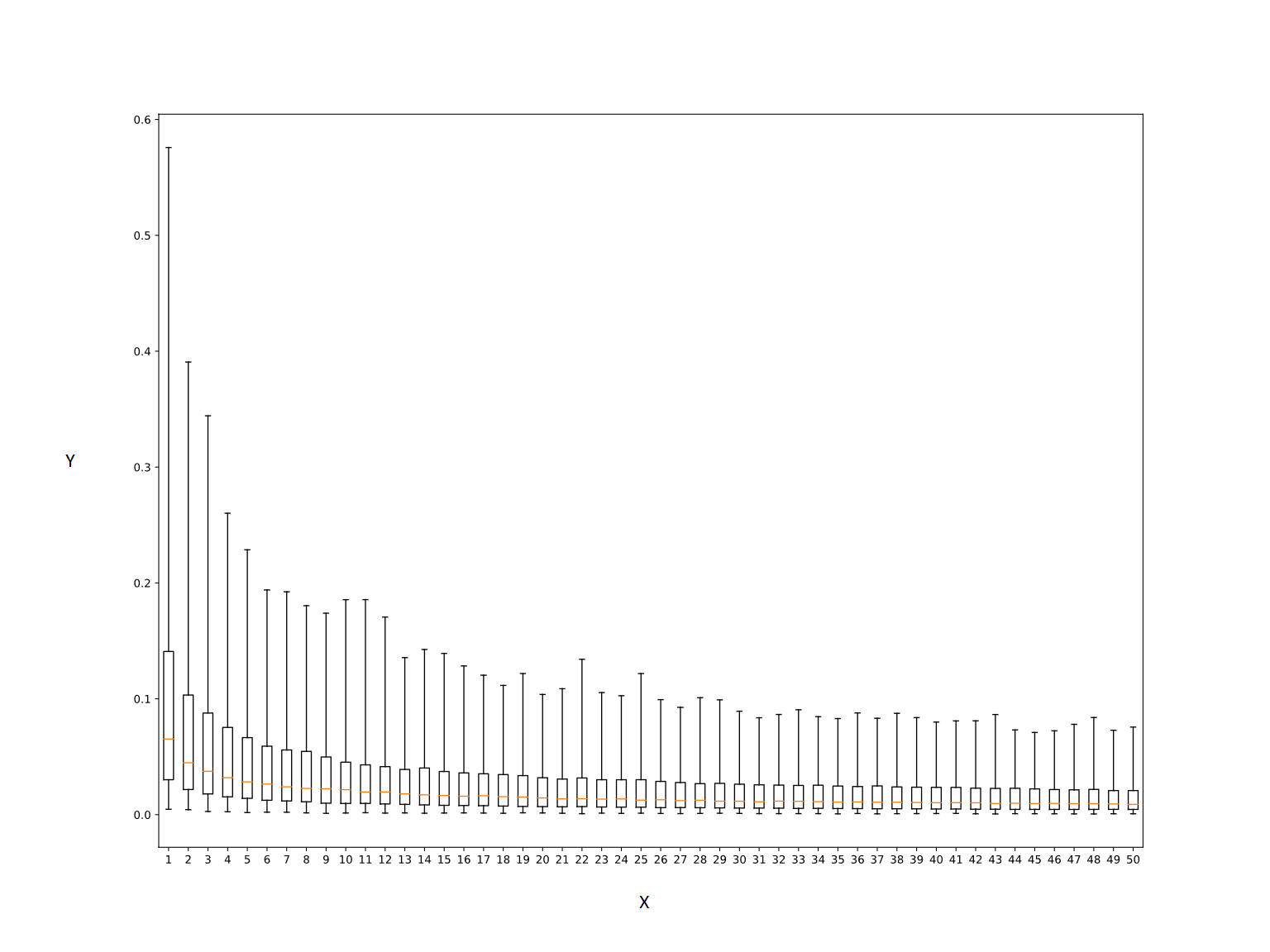

Supplement: Supplementary file 1 — Supplementary material 1 (png 39 KB) [file 11571_2021_9670_MOESM1_ESM.png]

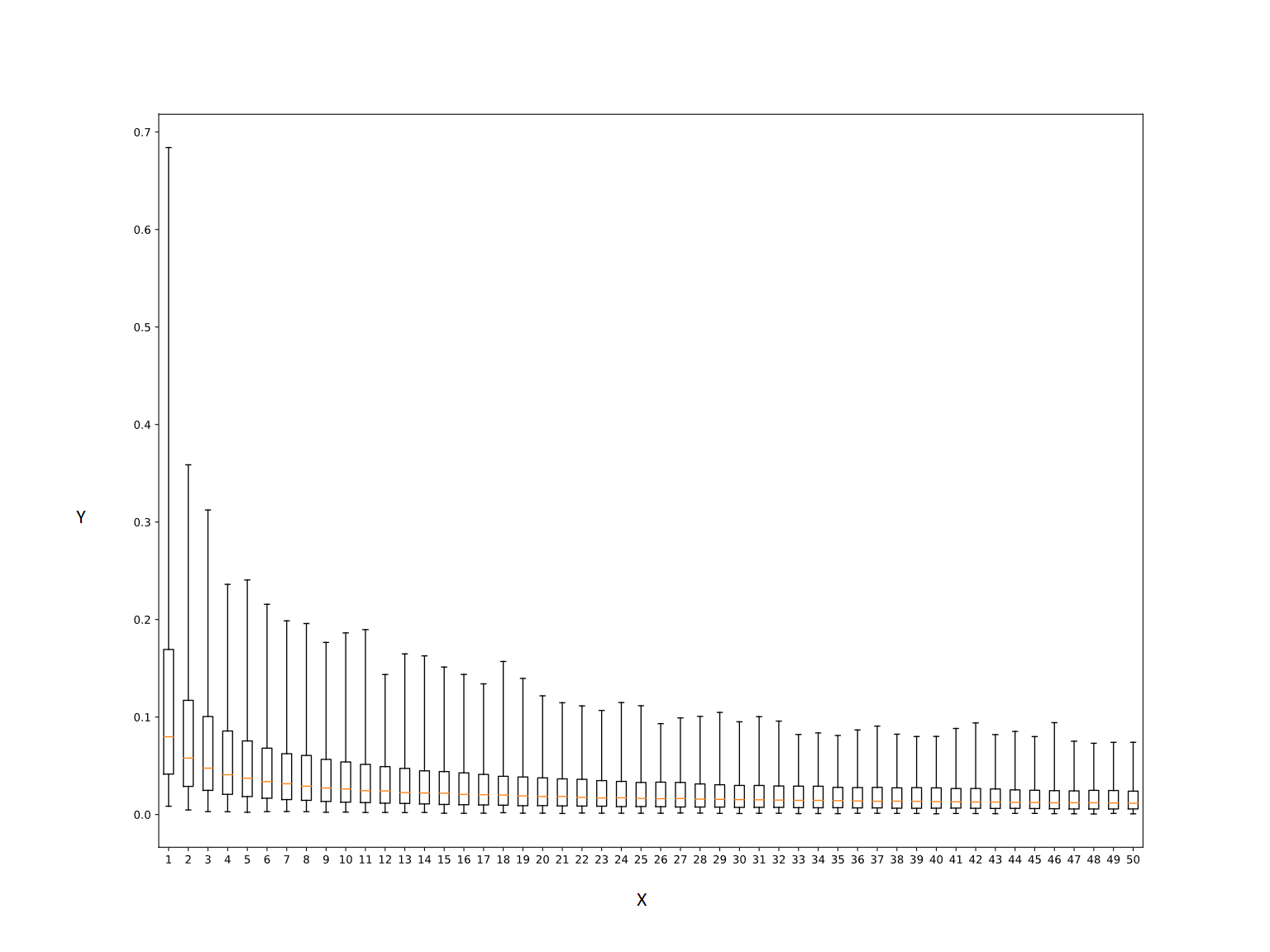

Supplement: Supplementary file 2 — Supplementary material 2 (png 39 KB) [file 11571_2021_9670_MOESM2_ESM.png]

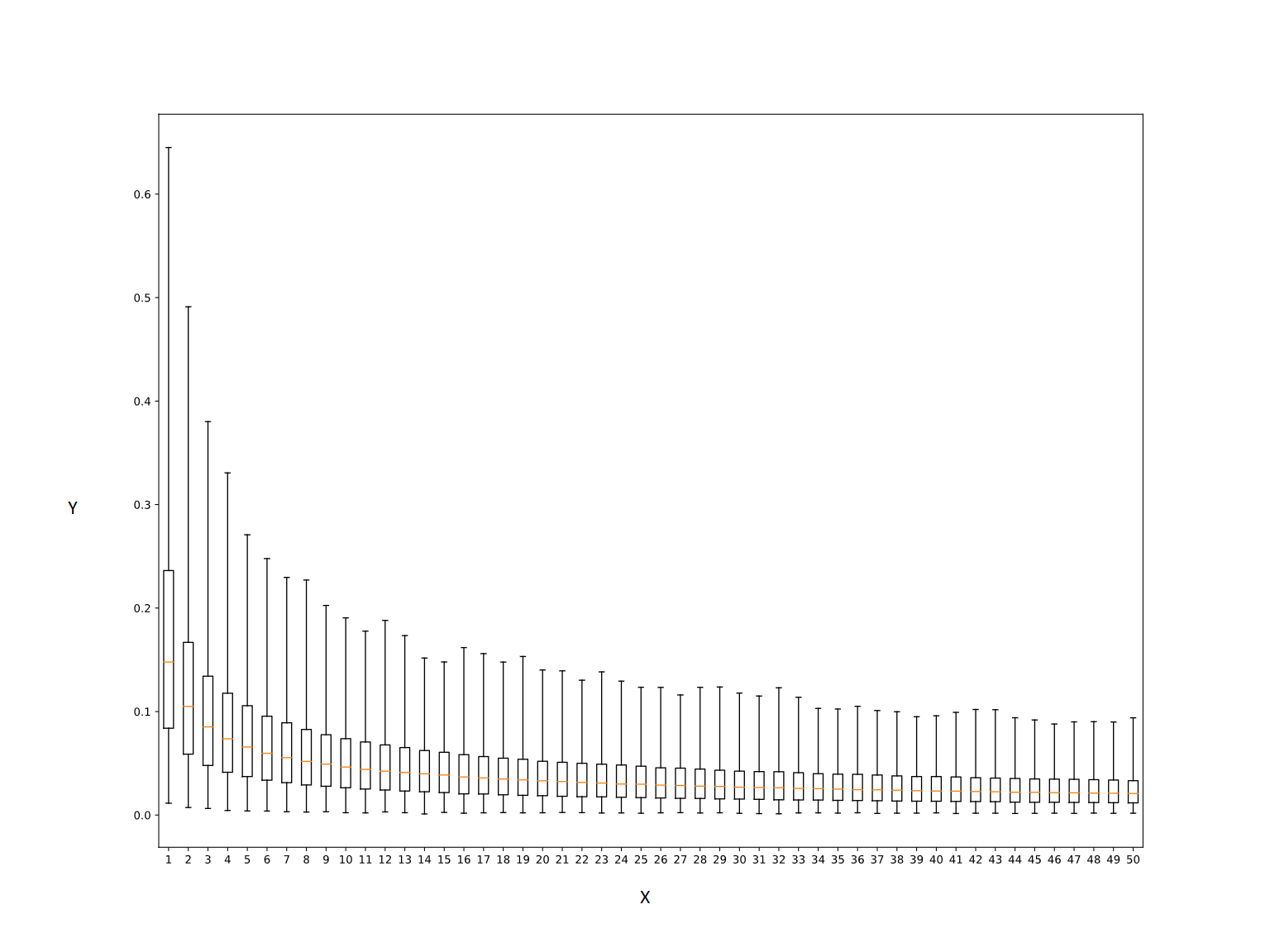

Supplement: Supplementary file 3 — Supplementary material 3 (png 39 KB) [file 11571_2021_9670_MOESM3_ESM.png]

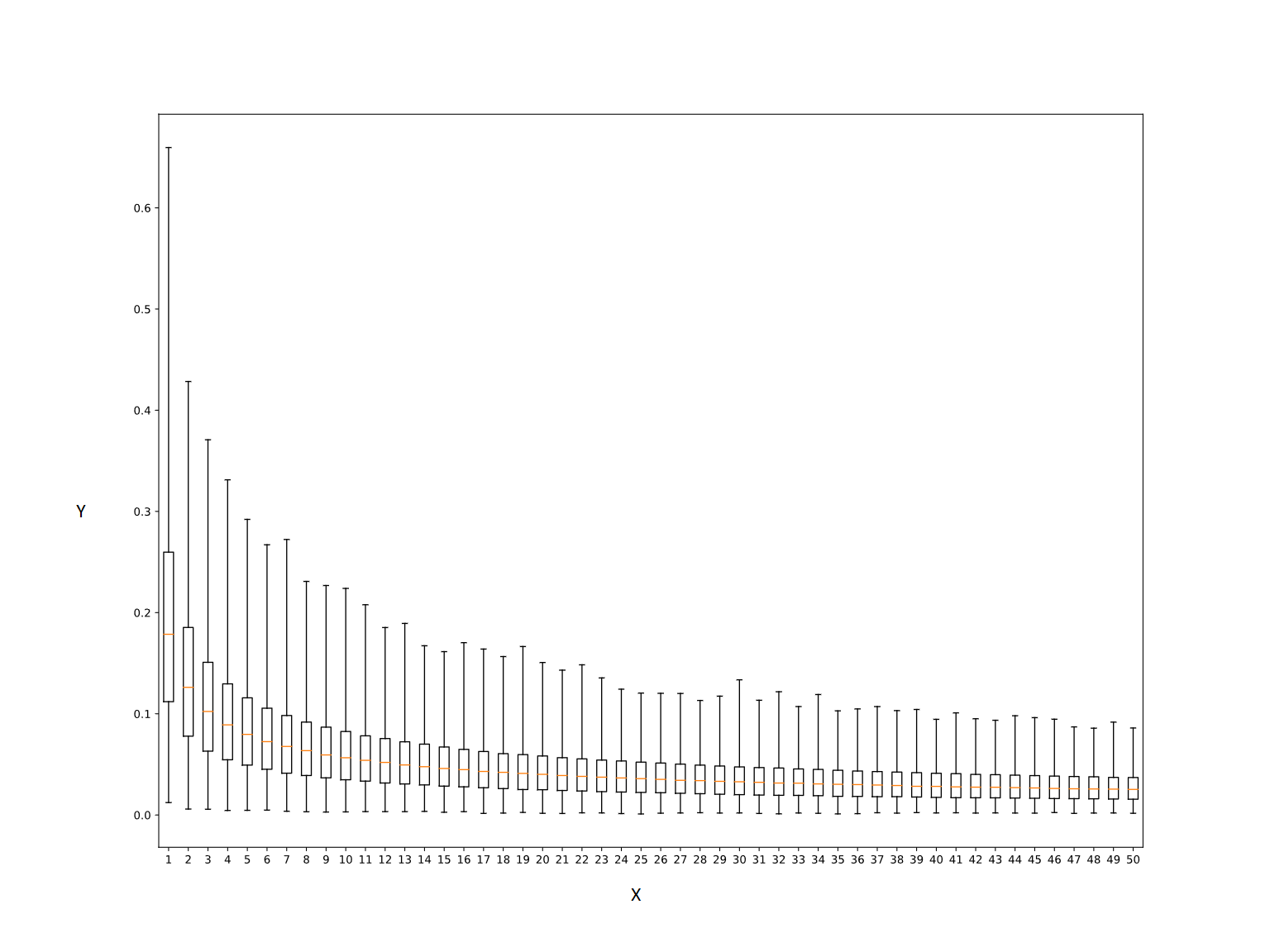

Supplement: Supplementary file 4 — Supplementary material 4 (png 40 KB) [file 11571_2021_9670_MOESM4_ESM.png]
